# Unveiling the Etiopathogenic Spectrum of Hypophysitis: A Narrative Review

**DOI:** 10.3390/jpm13081210

**Published:** 2023-07-30

**Authors:** Sara Menotti, Antonella Giampietro, Salvatore Raia, Miriam Veleno, Flavia Angelini, Tommaso Tartaglione, Simona Gaudino, Francesco Doglietto, Laura De Marinis, Alfredo Pontecorvi, Antonio Bianchi, Sabrina Chiloiro

**Affiliations:** 1 Pituitary Unit, Department of Endocrinology and Metabolism, Fondazione Policlinico Universitario A. Gemelli, IRCCS, 00168 Rome, Italy; sara.menotti@guest.policlinicogemelli.it (S.M.); antonella.giampietro@policlinicogemelli.it (A.G.); salvatore.raia@guest.policlinicogemelli.it (S.R.); miriam.veleno@guest.policlinicogemelli.it (M.V.); flavia.angelini@unicatt.it (F.A.); laura.demarinis@policlinicogemelli.it (L.D.M.); ipofisi@policlinicogemelli.it (A.P.); sabrina.chiloiro@unicatt.it (S.C.); 2 Department of Translational Medicine and Surgery, Università Cattolica del Sacro Cuore, 00168 Rome, Italy; tommaso.tartaglione@policlinicogemelli.it (T.T.); simona.gaudino@policlinicogemelli.it (S.G.); francesco.doglietto@policlinicogemelli.it (F.D.); 3 Department of Radiodiagnostic, Fondazione Policlinico Universitario A. Gemelli, IRCCS, 00168 Rome, Italy; 4 Department of Neurosurgery, Fondazione Policlinico Universitario A. Gemelli, IRCCS, 00168 Rome, Italy

**Keywords:** hypophysitis, immune checkpoint inhibitors, autoimmune

## Abstract

Hypophysitis, a rare inflammatory disorder of the pituitary gland, has seen an uptick in reported cases in recent years. Our objective is to summarize the most recent research on the etiopathogenesis, molecular mechanisms, and genetics of both primary and secondary hypophysitis. Primary autoimmune hypophysitis (PAH): During the acute phase of the disease, the pituitary gland in enlarged due to the infiltration of T and B lymphocytes. The chronic phase is characterized by progressive and irreversible pituitary atrophy. APA may play a role in the management, diagnosis, and prognosis of PAH. Specific autoantibodies such as anti-GH, anti-PIT-1, and anti-T-PIT have been found in patients with hypophysitis and hypopituitarism. A recent study suggested that a mechanism of escaping clonal deletion and mounting an immune response against self antigens can explain the unusual nature of the immune response observed in PAH patients. A cytokine array shows the presence of gamma-interferon and interleukin-17. Patients carrying mutations in the PIT1 or PROP1 genes may present PAH. Individuals carrying the HLA DQ8 haplotype are four times more likely to develop PAH. Immune checkpoint inhibitors induce hypophysitis (IIHs): IIHs is an increasingly frequent toxicity of in patients on treatment with inhibitors targeting cytotoxic T-lymphocyte antigen 4 (CTLA-4) and programmed cell death-1 (PD-1). ICIs inhibit the CTLA-4 pathway, leading to overactivation of T lymphocytes. The binding of PD-1/PD-L1 suppresses the activity of T cells, promotes the conversion of T-helpers into T-regulatory cells, and activates pro-survival signaling pathways in cancer cells. Cytokines play a crucial role in IIHs. B-cell infiltration has been observed in IIHs, suggesting that antibody-mediated pituitary injury may contribute. Genetic polymorphisms of CTLA-4 and PD-1 genes can increase the risk of IIHs. HLA alleles may also be involved in the onset of IIHs; this HLA association presents a possible alternative mechanistic hypothesis. IIHs may also be linked to a paraneoplastic syndrome triggered by ectopic expression of pituitary specific antigens. SARS-CoV-2-related hypophysitis: Recently, the literature has reported occurrences of hypophysitis associated with the SARS-CoV-2 virus; long COVID-19 may also present as infundibulo-neuro-hypophysitis. The virus enters the central nervous system because of its distinct interaction with angiotensin-converting enzyme receptors via spike proteins binding the capillary endothelium, and it directly damages the pituitary cells. The effect of SARS-CoV-2 can occur indirectly through inflammation and the release of cytokines. The exact mechanism remains ambiguous. The available data on endocrine complications associated with the SARS-CoV-2 vaccine are scant. Nonetheless, isolated cases of hypophysitis have been documented. Treatment of hypophysitis: Glucocorticoids are the cornerstone in managing primary hypophysitis, given their targeted action on inflammation. A better understanding of the etiopathogenesis and molecular mechanism of hypophysitis can lead to more effective and personalized treatment strategies.

## 1. Introduction

Hypophysitis, a rare inflammatory disorder of the pituitary gland, has seen an uptick in reported cases in recent years. This surge can be attributed to intensified research into the etiological causes of hypopituitarism, enhancements in radiological techniques, and the clinical use of drugs that may favor the occurrence of immune-related adverse events [1,2,3,4,5].

Hypophysitis exhibits significant heterogeneity and variability in its etiology. The most prevalent form is primary autoimmune hypophysitis (PAH), which is often idiopathic [4,5]. Moreover, hypophysitis has been identified as a clinically significant endocrine toxicity in patients undergoing treatment with immune-checkpoint inhibitors (ICIs). ICIs, by bolstering immune tumor surveillance, are approved for the treatment of various malignancies [1,2]. Furthermore, hypophysitis also appears to be prompted by a paraneoplastic autoimmune reaction. The presence of diverse ectopic proteins expressed in tumors can induce the formation of autoantibodies and autoreactive cytotoxic T cells [6,7,8]. Lastly, the recent literature has reported cases of hypophysitis linked to the SARS-CoV-2 virus, as well as to COVID-19 vaccination [5,9,10,11,12].

While the clinical aspects, diagnosis, and management of hypophysitis have been extensively studied, the etiopathogenesis and molecular mechanisms underlying hypophysitis remain largely elusive [4,5,13,14,15]. Acquiring a deeper understanding of the etiopathogenesis and discovering biomarkers for hypophysitis could potentially improve clinical practice by guiding more efficacious therapeutic choices [15].

In this narrative review, our objective is to summarize the most recent research on the etiopathogenesis, molecular mechanisms, and genetics of both primary and secondary hypophysitis.

## 2. Methods

We conducted an English language literature search on the topic “etiopathogenesis of hypophysitis”. We performed an electronic search using MEDLINE (PubMed database) in June 2023 using database-specific keywords: “hypophysitis” AND “pathogenesis”; “hypophysitis” AND “etiology”; “Primary hypophysitis”; “secondary hypophysitis”; “hypophysitis” AND “immune checkpoint inhibitors”; “hypophysitis” AND “anti-CTLA4”; “hypophysitis” AND “anti-PD-1”; “hypophysitis” AND “COVID-19”; “hypophysitis” AND “vaccination”; “hypophysitis” AND “genetics”; hypophysitis” AND “molecular”; “hypophysitis” AND “therapy”; “hypophysitis” AND “management”; “hypophysitis” AND “personalized”.

## 3. Primary Autoimmune Hypophysitis

### 3.1. Pathogenesis of Primary Autoimmune Hypophysitis

Autoimmune diseases are characterized by the body’s immune system attacking its own tissues and organs, leading to inflammation and tissue damage. PAH is a disease that targets the pituitary gland [16]. The pituitary gland is a highly vascularized peripheral organ located outside the blood–brain barrier, and it is supplied by both arterial and venous blood systems, as well as a venous portal system [17]. The endothelial cells surrounding the pituitary sinusoids form a barrier to the passage of secreted proteins from endocrine cells to the bloodstream. However, secreted proteins can still rapidly drain through the cavernous sinus to the jugular veins. Additionally, releasing hormones secreted from hypothalamic nerve terminals enter capillaries in the external zone of the median eminence, where they are collected and delivered via the hypophysial portal veins to the anterior pituitary gland. These hormones can also act as antigens of hypophysitis [2].

The exact etiology and pathogenesis of PAH are not fully understood, but studies using experimental autoimmune hypophysitis (EAH) in female mice have shed some light on the disease’s pathogenesis [18]. EAH was induced by immunizing female mice with extracts of whole pituitary gland. This experimental model showed that hypophysitis follows a biphasic natural history, with an acute phase followed by a chronic phase. During the acute phase of the disease, the pituitary gland is enlarged due to the infiltration of T and B lymphocytes and plasma cells. In contrast, the chronic phase is characterized by progressive and irreversible pituitary atrophy due to the fibrotic process. It is interesting to note that, in SJL/J mouse models, the chronic phase of EAH is associated with a reduction in the plasmatic concentration of anti-pituitary antibodies (APA) and pituitary hormones [19].

**Figure 1:**
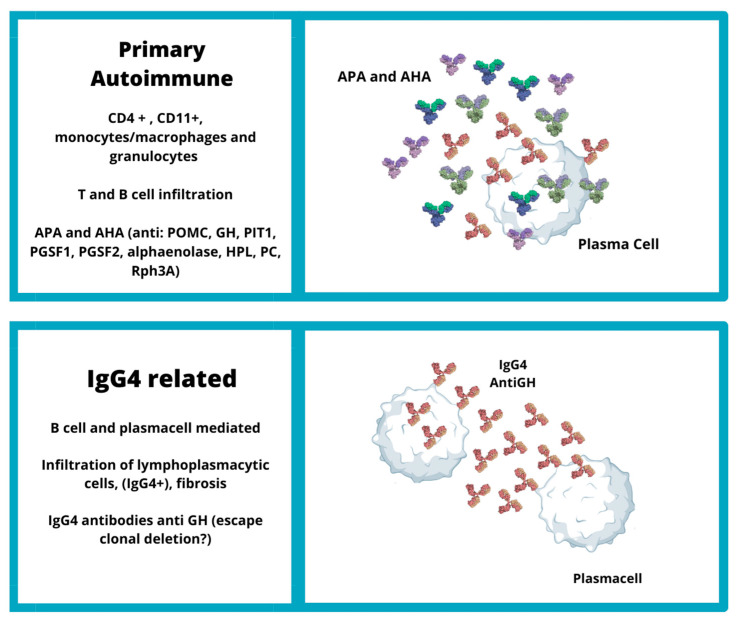
Etiopathogenesis of primary autoimmune hypophysitis and IgG4-related hypophysitis. (CD: cluster of differentiation; Th: T helper cells; APA: anti-pituitary antibodies; AHA: anti-hypothalamus antibodies; ACTH: adrenocorticotropic hormone; Pit1: pituitary transcription factor 1; IL17: interleukin 17; IFN-γ: interferon-gamma; POMC: pro-opiomelanocortin; POMC: pro-opiomelanocortin; PGSF: pituitary gland specific factor; HPL: anti-chorionic somatomammotropin hormone; PC: anti-prohormone convertase; Rph3A: rabphilin-3A; IgG4: immunoglobulin G4; GH: growth hormone).

The extent of lymphocytic infiltration corresponds to the degree of pituitary gland enlargement, and partial or complete hypopituitarism may occur if the pituitary tissue is destroyed due to fibrosis or extensive lymphocytic infiltration. Partial or complete hypopituitarism may resolve if pituitary gland tissue is not destroyed. As the disease progresses, fibrosis of the pituitary gland may also occur, with irreversible hypopituitarism [20] (Figure 1).

### 3.2. Classification of Hypophysitis

Hypophysitis can be categorized anatomically; adeno-hypophysitis affects the anterior portion of the gland: infundibuloneurohypophysitis pertains to the posterior, and panhypophysitis involves the entire gland [21,22]. In rarer cases, the inflammation can spread into the hypothalamus or may manifest as isolated hypothalamitis [4,23].

Moreover, hypophysitis can be classified histologically into various forms: lymphocytic, granulomatous, xanthomatous, IgG4-related, and necrotizing hypophysitis, as well as mixed forms (lymphogranulomatous and xanthogranulomatous) [4,23] (Table 1).

Lymphocytic hypophysitis (LH) represents the most frequent form of hypophysitis, accounting for approximately two-thirds of all cases. LH predominantly affects women, particularly during the late stages of pregnancy or the immediate postpartum period, with a primary impact on the anterior pituitary. A common symptom of LH is adrenocorticotrophic hormone (ACTH) deficiency. The speculated link between hypophysitis and pregnancy pertains to the increased pituitary hyperplasia during pregnancy, leading to enhanced pituitary antigens and changes in pituitary blood flow. This results in increased pituitary blood supply and greater exposure to the immune system [4,5]. The correlation between LH and autoimmunity is evident, given its association with personal or familial history of autoimmune diseases and specific HLA DQ8 and DR3 alleles [4]. LH is believed to be a T-cell-mediated event; in a murine model, T-cell activation, rather than autoantibody/B cell infiltration, was observed. CD4^+^ T cells are predominant and exhibit a specific phenotype with T helper 17 and 1 cells, respectively producing interleukin 17 (IL17) and interferon-gamma (IFN-γ) [24]. From a histopathological perspective, there is significant infiltration of lymphocytes, mainly T cells, into the interstitium, complemented by B cells, plasma cells, and occasional eosinophils, macrophages, histiocytes, and mast cells. If not treated in a timely manner, fibrosis is the typical outcome [5,25].

Granulomatous hypophysitis typically manifests in women during their fourth decade of life and often presents as a local symptom of diverse and widespread multisystem diseases. These can include sarcoidosis, Wegener’s granulomatosis, Crohn’s disease, Takayasu arteritis, Cogan’s syndrome, tuberculosis, and other vasculites [5,26,27,28]. Granulomatosis with polyangiitis hypophysitis is rare (<1%) [4]. The first description of idiopathic granulomatous hypophysitis was provided by Brissaud in 1908 and further detailed by Simmonds in 1917. Following these initial descriptions, several individual case reports and a few case series have been published [28]. Histologically, granulomatous hypophysitis is characterized by the formation of non-necrotizing granulomas, comprising epithelioid histiocytes, multinucleated giant cells, and a surrounding lymphocyte infiltrate [4,5,27]. Debates persist regarding the pathogenesis of idiopathic granulomatous hypophysitis. One significant point of contention is whether LH and granulomatous hypophysitis are separate diseases or are time-dependent manifestations of the same disease. It is proposed that granulomatous hypophysitis could represent a later stage of inflammatory hypophyseal disease than LH, potentially occurring in a subset of patients who initially developed subclinical LH. Interestingly, in a mouse model of LH, some test animals developed multinucleated giant cells similar to those seen in granulomatous hypophysitis [17,28]. Additionally, evidence from the literature suggests that APA may also have a role in granulomatous hypophysitis. This implies that there might be multiple autoimmune targets within the pituitary gland, each capable of triggering different and overlapping forms of pituitary inflammation [28].

Certain specific instances of granulomatous hypophysitis are associated with histiocytosis, a range of diseases derived from abnormal Langerhans cells, which are dendritic or antigen-presenting cells. This spectrum includes Langerhans cell histiocytosis and Erdheim–Chester disease. The hypothalamic–pituitary region is infiltrated in 5–50% of all patients with Langerhans cell histiocytosis, most commonly in those presenting the multifocal form of the disease. Isolated occurrences, however, are quite rare [29]. This infiltration predominantly affects the neurohypophysis, leading to diabetes insipidus as the most common initial symptom. Histologically, the disease shows granulomatous involvement of monoclonal Langerhans cells (CD1a^+^ and CD207^+^), alongside polyclonal inflammatory cells, particularly T lymphocytes, macrophages, and eosinophils. In the case of Erdheim–Chester disease, histiocytes are positive for CD68 and negative for CD1a [4,29].

Xanthomatous hypophysitis (XH), a rare variant, is typically cystic in nature. This histopathological form is characterized by its lipid-rich, foamy histiocytes. Representing 3% of all panhypophysitis cases, XH is more frequently observed in women than men [4,5,22]. The pathogenesis is typically attributed to an inflammatory response prompted by exposure to elements of a Rathke cleft cyst after rupture, hemorrhage, or leakage [5]. This initial trigger causes a secondary response to the mucoid fluid content released from the cyst. As a result, the ensuing inflammation becomes a chronic process, attracting macrophages and accumulating lipids [30]. In terms of gross appearance, XH results in a pituitary cyst filled with an orange or amber-colored thick fluid and crystal-like structures resembling pus. Although the condition can affect the posterior pituitary and hypothalamus, it primarily involves the anterior region [30]. From a histological perspective, a mixed inflammatory infiltrate composed of lipid-laden histiocytes, resembling xanthomas, is observed. XH and xanthogranulomatous hypophysitis represent a continuum with overlapping clinical and pathological features. Xanthogranulomatous lesions exhibit cholesterol clefts, fibrosis, giant cells, eosinophilic necrotic debris, macrophage accumulation, and hemosiderin deposits. In contrast, xanthomatous lesions lack hemosiderin pigmentation. This implies that the xanthomatous form might transition into the xanthogranulomatous form, potentially as a chronic process following repeated Rathke cleft cyst hemorrhages [5,31].

Necrotizing hypophysitis represents the least common histological variant, accounting for approximately 0.6% of all panhypophysitis cases. Given its rarity, only a handful of cases have been reported in the literature, and its pathogenesis remains largely unclear [4,5]. The disease is characterized by extensive tissue necrosis, fibrinoid degeneration, and the infiltration of a mixture of inflammatory cells. Necrotizing hypophysitis can potentially cause symptoms reminiscent of pituitary apoplexy [5,27].

IgG4-related hypophysitis, a plasma cell variant, is categorized as primary hypophysitis. It may present either as an isolated pituitary lesion or as part of a multisystemic disease. The IgG4-related disease can affect numerous organs, leading to a variety of clinical manifestations, the most common are retroperitoneal fibrosis, sclerosing sialadenitis, adenopathy, and pancreatitis. Pituitary involvement in IgG4-related disease affects 4–5% of patients. Interestingly, 15–25% of patients, predominantly women, will exhibit normal serum IgG4 levels [4,32]. The pathogenesis of IgG4-related hypophysitis is not fully understood, although an association with the BRAF V600E mutation has been reported [33]. The role of IgG4 antibodies in disease pathogenesis is yet to be definitively established, and it is uncertain whether they are involved in the causative pathway or are merely benign bystanders. In 2011, Salgado et al. identified two pituitary targets recognized by serum antibodies in a patient with IgG4-related hypophysitis: growth hormone (GH) and pro-opiomelanocortin (POMC). The pathogenic hypothesis is based on the presence of lymphocytes reactive to GH variants that can evade immune tolerance. Therefore, novel GH epitopes, normally hidden from the immune system, may be generated in an inflammatory environment and become dominant [32,34]. Histologically, IgG4-mediated hypophysitis is marked by an abundant infiltration of lymphoplasmacytic cells, a significant number of IgG4-positive plasma cells, and a storiform pattern of fibrosis [4,32] (Figure 1).

### 3.3. Antibodies in Primary Autoimmune Hypophysitis

Preliminary data suggest that the identification of APA may play a role in the management, diagnosis, and prognosis of PAH. Various methods have been used to investigate APAs, including immunofluorescence, radioligand binding assays, and the complement consumption test [35,36]. The most effective substrate for APA detection in immunofluorescence testing was found to be baboon pituitary [37]. The method utilizes serum APAs that bind to corresponding antigens in the pituitary sections, with the resulting antigen–antibody complexes detected using FITC conjugated with goat anti-human IgG. Positive cases were characterized by a diffuse immunofluorescence pattern and an intracytoplasmic staining, typically seen at a dilution rate of 1:8 [38]. APAs were previously known as the only molecular biomarkers for hypophysitis, yet recent research showed that APAs are present in other autoimmune disorders of the pituitary gland [36,39,40]. Furthermore, recent studies have reported a significantly higher prevalence of APAs in patients with PAH (68.4%) compared to patients with non-secreting pituitary adenomas (22%) or healthy controls (14%) [41]. It has also been found that the simultaneous positivity for anti-pituitary and anti-hypothalamus antibodies is rare in patients with non-secreting pituitary adenoma, but present in 52.9% of patients with PAH [41]. APA testing is an essential tool for the diagnosis, management, and prognostication of PAH. The detection of APAs is particularly important in predicting the response to glucocorticoid treatment in PAH [42]. Although the clinical relevance of APAs is evident, their diagnostic value is still debated in previous studies. While APAs have been considered a pathogenic marker of hypophysitis, recent research suggests that they may also be clinically helpful in diagnosing acute hypophysitis in humans, but only if detected at high concentrations [39].

### 3.4. Autoantigens of Primary Autoimmune Hypophysitis

The identification of the autoantigens that are involved in PAH is essential for understanding the pathophysiology of this disease. Lupi et al. reported that whole mouse pituitary extracts and cytosol fractions are more immunogetic than pituitary membranes and nuclei, and that a high immunogen dose correlated with more severe hypophysitis [18]. Specific autoantibodies such as anti-alpha enolase, anti-GH, anti-PGSF1a and 2, anti-chorionic somatomammotropin hormone (HPL), anti-prohormone convertase (PC), anti-PIT-1, anti-POMC, anti-alpha rad guanine nucleotide dissociation inhibitor, anti-secretogranin, anti-TDRD6, and anti-T-PIT have been found in patients with hypophysitis and hypopituitarism [36,43,44]. The growth hormone and pro-opiomelanocortin have also been suggested as antigens of IgG4-related hypophysitis [45]. Antibodies against GH, PGSF1a, PGSF2, and T-PIT have been detected in healthy controls and in patients with isolated ACTH deficit or other autoimmune diseases [7,43]. Additionally, rabphilin-3A (Rph3A) has been described as a putative antigen of infundibulo-neuro-hypophysitis [46,47]. The dual nature of the gland due to its embryogenesis may explain the different antigenic profile in infundibulo-neuro-hypophysitis. 

During uterine development, adenohypophysis derives from epithelial cells and is composed of the pars distalis, a thin layer called the pars tuberalis, and the pars intermedia, while the neurohypophysis develops from a downgrowth of neural tissue at the base of the diencephalon and gives rise to the pars nervosa, the infundibulum, and the median eminence. The infundibulum and the pars tuberalis make up the pituitary stalk [48]. This different embryogenic origin of the adeno-pituitary and the neuro-pituitary may also explain the different inflammatory involvement that occurs in adeno-hypophysitis, infundibulo-neuro-hypophysitis, and pan-hypophysitis. In adeno-hypophysitis, the inflammatory process only involves the adeno-pituitary, whereas in infundibulo-neuro-hypophysitis, the pituitary stalk, and the neuro-pituitary are involved. In cases of pan-hypophysitis, all of these structures are involved [16]. In a recent study, a specific reactivity to ALDOA was found in PAH patients by ELISA test. This discovery paves the way for ALDOA to potentially be a valuable indicator of autoimmune damage in these patients. However, reactivity for GH, SOD2, and ACTB antigens was similar between PAH patients and normal donors. This observation hints at the existence of T and B cells that are either specific to self-antigens or exhibit cross-reactivity with different antigens. GH is the only antigen among the investigated ones that is exposed to the immune system in physiological conditions. Therefore, the immune system is expected to be familiar with GH and to not react against it. The B cells specific for intracellular proteins often escape clonal deletion (the immune system’s self-tolerance mechanism of apoptosis that eliminates B cells that can potentially harm body tissues). Therefore, novel GH epitopes, normally cryptic to the immune system, can be generated in an inflammatory response and become dominant [34]. Therefore, this mechanism of escaping clonal deletion and mount immune response against self-antigens (e.g., GH), further underscores the unusual nature of the immune response observed in PAH patients [49]. The idea that GH could act as an autoantigen is also supported by the fact that growth hormone deficiency is often found in individuals with pituitary antibodies [34].

In terms of POMC as an autoantigen, pro-opiomelanocortin is mainly expressed in the pituitary gland and skin, but is also present in lower levels in other tissues, including the placenta. During pregnancy, POMC becomes detectable in plasma and then rapidly decreases postpartum, becoming undetectable within 3 days after delivery [50]. This could offer an explanation for the temporal association between autoimmune hypophysitis and pregnancy [35]. A mechanism similar to the one proposed for growth hormone could occur with POMC, where an immune reaction against placental-derived POMC could extend to the pituitary. Considering that ACTH deficiency is the most common endocrine abnormality reported in patients with hypophysitis POMC is an attractive candidate autoantigen [51].

### 3.5. Cell-Mediated Immune Response in Primary Autoimmune Hypophysitis

The cell-mediated immune response in PAH has not been extensively researched, but studies in SIL/J murine models have provided some insights. Cytometry studies proved that the number of hematopoietic cells increased in immunized mice compared to nonimmunized mice, accounting for about 85% of all cells in pituitary extracts of immunized mice, while they accounted for only 2% in nonimmunized mice. CD4-positive T-lymphocytes, with an activated/memory phenotype, were three times more abundant than CD8-positive T lymphocytes in the SIL/J mice [19]. In addition, monocytes/macrophages and granulocytes were also detected in SIL/J experimental hypophysitis. Dendritic CD11-positive cells were identified in close proximity to the lymphocyte aggregate, suggesting their role in presenting antigens to infiltrating T cells and stimulating cytokine secretion. Cytokine array membranes on pituitary extracts of SIL/J hypophysitis showed the presence of gamma-interferon and interleukin-17, while immunohistochemical studies revealed active T- and B-cell proliferation in the pituitary gland [52]. Our recent study also showed that neutrophil and lymphocyte counts and the neutrophil/lymphocyte ratio were interchangeable in a cohort of 19 PAH cases and 50 healthy controls [41]. 

### 3.6. The Genetics of Primary Autoimmune Hypophysitis

Hypophysitis is a disorder whose genetic basis is still not fully understood due to its complex etiology and the possible involvement of other causes of hypopituitarism, such as congenital diseases that lead to abnormal pituitary gland development. Patients carrying mutations in the PIT1 or PROP1 genes may present with this disorder [53,54], making it necessary to differentiate child-onset hypopituitarism from pediatric hypophysitis and pituitary hyperplasia. The presence of certain human leukocyte antigen (HLA) polymorphisms has been described in patients with PAH. In one study, HLA-DR4 and HLA-DR5 were found in 44% and 23% of cases, respectively, between 1987 and 1999 [55,56]. More recently, HLA haplotypes DQ8 and DR53 were identified in 87% and 80% of cases, respectively, in a series of 15 PAH patients [56]. A recent study identified the presence of 12 HLA haplotypes associated with celiac disease in 16 consecutive Caucasian patients affected by PAH, with the prevalence of the DQ8 haplotype being 25% [57]. This result was significantly higher than the 7.2% observed in a control group of 250 consecutive Caucasian individuals. Furthermore, individuals carrying the DQ8 haplotype were four times more likely to develop PAH, indicating the presence of the DQ8 haplotype as a susceptibility factor for hypophysitis, similar to its association with celiac disease. Including HLA-DQ8 molecular testing in the diagnostic flowchart of PAH could help physicians in the differential diagnosis of focal pituitary lesions. 

The HLA system is modular in organization, with some genetic loci (beta-DQ, alpha-DQ, DRB1, DRB2, and DRA) located in close proximity to each other. HLA is a gene cluster on the sixth chromosome, containing more than 200 coding genes that encode cell-surface proteins responsible for regulating the immune system. In humans, three classes of HLA have been identified: HLA class I genes (A, B, and C), HLA class II genes (DP, DQ, and DR), and HLA class III genes (those of complement factors C2 and C4, and TNF) [58]. Class II HLA alleles are primarily involved in autoimmune disease. There are over 7000 different alleles in HLA, and approximately 2000 of these alleles belong to the DR gene. Beta-DQ has around 165 alleles, and their combination produces about 116 different proteins. In any individual’s genetic makeup, different maternal and paternal alleles combine for different genetic loci (DRB, HLA-B, HLA-A, etc.), resulting in the formation of different major histocompatibility complexes (MHCs) that encode glycoproteins. These glycoproteins are expressed only on macrophages, B cells, and dendritic cells, and their primary function is to present antigens to competent cells, such as T-helper lymphocytes, determining the immunological response [58]. The HLA system plays a crucial role in the immune response, and its dysfunction has been associated with various autoimmune diseases.

## 4. Immunotherapy Induced Hypophysitis

### 4.1. Pathogenesis of Immunotherapy Induced Hypophysitis

Hypophysitis is an increasingly frequent endocrine toxicity of clinical significance observed in patients on treatment with ICIs, particularly monoclonal antibodies (mAbs) that target cytotoxic T-lymphocyte antigen 4 (CTLA-4) and programmed cell death-1 (PD-1) [1,2]. Nonetheless, there are limited data available on the molecular mechanism underlying this disorder.

The varying incidence rates of ICI-induced hypophysitis (IIH) have been attributed to the different mechanisms of action of the ICI: antibodies that block CTLA-4 enhance T-cell priming, while those that block PD-1/PD-L1 seem to enhance an existing CD8 T-cell response [2,59]. 

**Figure 2:**
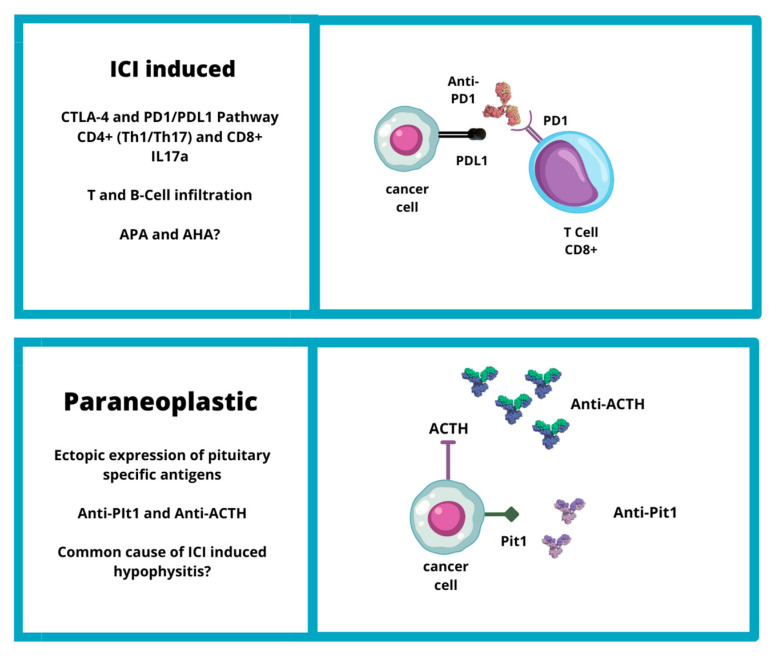
Etiopathogenesis of IIH (CTLA4: cytotoxic T-lymphocyte-associated protein 4; PD1: programmed cell death protein-1; PDL1: programmed death-ligand 1; ACTH: adrenocorticotropic hormone; Pit1: pituitary transcription factor 1).

CTLA-4 is expressed on the cell surface of active CD4-positive and CD8-positive T cells. The primary function of the CTLA-4 pathway is to drain lymph nodes where naïve T-cells are primed by exposure to tumor antigens (presented by antigen-presenting cells) and become activated [60]. CTLA-4 binds CD80 and CD86, which are expressed on the cell surface of APCs, with higher affinity and avidity than CD28 [61]. The engagement of CTLA-4 with CD80 and CD86 mitigates the immune response [62]. ICIs inhibit the CTLA-4 pathway, leading to overactivation of T-lymphocytes. This overactivation may predispose to the onset of autoimmune disease, such as IIH. The increased incidence of hypophysitis in patients treated with anti-CTLA-4 has also been linked to the potential ectopic expression of CTLA-4 at the pituitary gland level. This ectopic expression may act as an autoantigen, which could trigger an autoimmune reaction by anti-CTLA-4 antibodies in cancer patients undergoing ICI treatment [59] (Figure 2). 

Regarding PD-1 targeted ICIs, PD-1 is an inhibitory receptor expressed mainly on activated CD8-positive T-lymphocytes [2,63]. PD-1 is triggered by PD-ligands 1 and 2 (PD-L1 and PD-L2, respectively), which are constitutively expressed on tumor cells [64,65]. The binding of PD-1/PD-L1 suppresses the activity of T-cells [66] promotes the conversion of T-helpers into T-regulatory cells [67] and activates pro-survival signaling pathways in cancer cells, leading to resistance to cytotoxic T-lymphocytes [68]. According on the inhibition of this mechanism, treatment with ICI targeting PD-1 and PD-L1 is associated with a high frequency of autoimmune disorders, including endocrine toxicity [1].

Recent findings suggest that cytokines, in addition to T-cell-mediated cytotoxicity, play a crucial role in IIHs. In mouse models of autoimmune hypophysitis, infiltrates of CD4 T cells with T helper cell 1 and 17 (Th1/Th17) and T helper cell 17 (Th17) cytokine profiles were observed in the pituitary gland. Furthermore, transcriptome analysis of IIHS demonstrated the predominant expression of interleukin 17A (IL-17A), CD4, and MHC class II antigens [69]. Chalan et al. isolated RNA from the formalin-fixed paraffin-embedded pituitary specimens of 16 hypophysitis patients (three of whom had IIHs). All three secondary hypophysitis patients showed detectable IL-17A levels and other cytokines were not detected [24].

B-cell infiltration has been observed in IIHs, suggesting that antibody-mediated pituitary injury may contribute to the pathogenesis of IIHs as in autoimmune hypophysitis [51,69]. The prevalence of APAs and AHAs during anti-PD-1/anti-PD-L1 therapy is higher than in healthy individuals; in some patients, APA/AHA positivity has been observed after just 9 weeks of immunotherapy [70]. APAs, measured by indirect immunofluorescence, are a surrogate marker of the presence of autoimmunity against the pituitary gland and are detected more frequently in some pituitary diseases, particularly biopsy-proven hypophysitis. Although APAs are negative at baseline, one study demonstrated that APAs became positive at the onset of ipilimumab (anti-CTLA-4 antibody)-induced hypophysitis in all examined patients [71].

Lastly, the prevalence of hypophysitis in patients treated with anti-PD-1 and anti-PD-L1 increases when these ICIs are associated with anti-CTLA-4 antibodies or when patients have pre-existing autoimmune or inflammatory disorders. One proposed mechanism is an increase in T-cell activity against shared tumor and normal tissue antigens due to pre-existing antibodies or inflammatory cytokines. However, studies on the detection of antibodies in cancer patients treated with ICIs, particularly PD-1 and PD-L1 inhibitors, are rare and have primarily focused on the detection of APA, not AHA. Recently, APA was detected in two out of four cancer patients with hypophysitis related to anti-PD-L1 or anti-PD-1 treatment [51,59].

### 4.2. Genetic Factors of ICI-Induced Hypophysitis

The pathogenesis of IIHs involves various mechanisms, among which genetic factors play a crucial role. It is well known that genetic polymorphisms of CTLA-4 and PD-1 genes can increase the risk of developing autoimmune diseases, including IIHs [72,73]. These polymorphisms may not alter the CTLA-4 amino-acid sequence but can affect the affinity for CTLA-4 mAbs, thereby increasing the risk of immunotherapy-induced autoimmune disorders [72].

HLA alleles may also be involved in the onset of IIHs, with several HLA haplotypes being associated with autoimmune diseases [8]. In particular, a Japanese study showed that HLACw12 and HLA-DR15 were significantly associated with anti-CTLA-4 related hypophysitis, whereas HLA-DQB106:01, HLA-DPB109:01, and HLA-DRB5*01:02 were significantly associated with anti-PD-1-related hypophysitis [69]. Another study found that HLA-Cw12, HLA-DR15, HLA-DQ7, and HLA-DPw9 were significantly more prevalent in patients with immunotherapy induced central hypoadrenalism [8,71]. This HLA association presents a possible alternative mechanistic hypothesis in contrast to the proposed hypothesis that ICIs hypophysitis is due to direct binding of CTLA-4 inhibitors to pituitary cells [74].

Additionally, copy number variations (CNVs) and small variations (VARs) may also be associated with the occurrence of IIHs. A study analyzing 95 melanoma patients treated with ICIs found that genes affected by VARs associated with hypophysitis include SMAD3, PRDM1, and IL1RN, while genes affected by CNVs related to the occurrence of hypophysitis are TERT, SMAD3, JAK2, PRDM1, FAN1, CD274, and UNG [70].

Lastly, congenital forms of isolated ACTH deficiencies commonly involve the T-PIT gene, also known as the TBX19 gene, and the POMC gene, which encodes the melanocortin protein [71].

### 4.3. Paraneoplastic Syndrome Hypothesis

Recently, a hypothesis has emerged that IIHs may be linked to a paraneoplastic syndrome triggered by ectopic expression of pituitary specific antigens. Two new clinical entities have been identified: anti-PIT-1 hypophysitis and paraneoplastic autoimmune isolated ACTH deficiency [6] (Figure 2). 

Bando et al. described the mechanism of a new kind of hypophisitis: anti-PIT-1 hypophysitis. It was described as paraneoplastic autoimmune hypophysitis due to ectopic expression of PIT-1 or pituitary hormones in complicated tumors that evokes autoimmunity against pituitary cells, leading to the production of anti-Pit-1 autoantibodies and autoreactive cytotoxic T cells. The presence of autoimmunity directed against PIT-1 could explain the pituitary hormone abnormalities in some cancer patients and that tumor tissues sometimes exhibited ectopic expression of pituitary antigens and hormones, such as POMC [6]. In addition, in an autopsy specimen, it was observed that the PIT-1 epitope presented with MHC class I antigen on the surface of patient pituitary cells [75]. Furthermore, the presence of a common underlying mechanism between “paraneoplastic autoimmune hypophysitis” and PD-1/PDL-1 inhibitor-related hypophysitis was suggested. Actually, some cases of PD-1/PD-L1 inhibitor-related hypophysitis could be caused by these paraneoplastic autoimmune mechanisms [6]. 

Similarly, in paraneoplastic autoimmune isolated ACTH deficiency, ectopic expression of cancer cells in ACTH can promote the immune response, leading to the synthesis of anti-ACTH antibodies that can also act on pituitary corticotroph cells and cause central hypoadrenalism [51]. Recent research suggests that IIHs and ACTH deficits may be considered two different endocrine toxicities with manifestation of the same clinical entity. A study conducted on 62 cancer patients treated with ICIs found that the prevalence of APAs was similar among the five patients who developed an IIH (APA positivity: 80%) and in those who developed an ACTH deficit (APA positivity: 88.2%) entity [8]. Kobayashi et al. (2021) reported that APAs recognized ACTH-secreting cells in all patients with positive APAs, not only at baseline but also at the onset of ICI-induced isolated ACTH deficiency. Moreover, in some patients, APAs recognized pituitary cells secreting other hormones such as TSH, FSH, LH, GH, and/or PRL at the onset of ICI-induced isolated ACTH deficiency [51,71].

## 5. SARS-CoV-2 and Vaccine-Related Hypophysitis

### 5.1. SARS-CoV-2-Related Hypophysitis

COVID-19 is known to induce endocrine abnormalities, both via an autoimmune mechanism and by causing damage to organs. The recent literature has reported occurrences of hypophysitis associated with the SARS-CoV-2 virus [11,12,76,77]. Several cases of diabetes insipidus have been noted in patients on the same week after COVID-19 infection [11,12]. Disruption of adeno-hypophyseal functions was corroborated by histological alterations correlated with disease severity in pituitary cells obtained from autopsies [78]. 

Long-COVID may also present as an infundibulo-neuro-hypophysitis. It was determined that 24 survivors of severe COVID-19 exhibited signs of hypocortisolism attributed to post-infectious hypophysitis [11]. In SARS patients’ survivors, 24 (39.3%) patients had evidence of hypocortisolism, with most cases resolving within a year, indicative of temporary hypothalamic–pituitary dysfunction due to either the use of exogenous steroids or direct viral damage [79].

**Figure 3:**
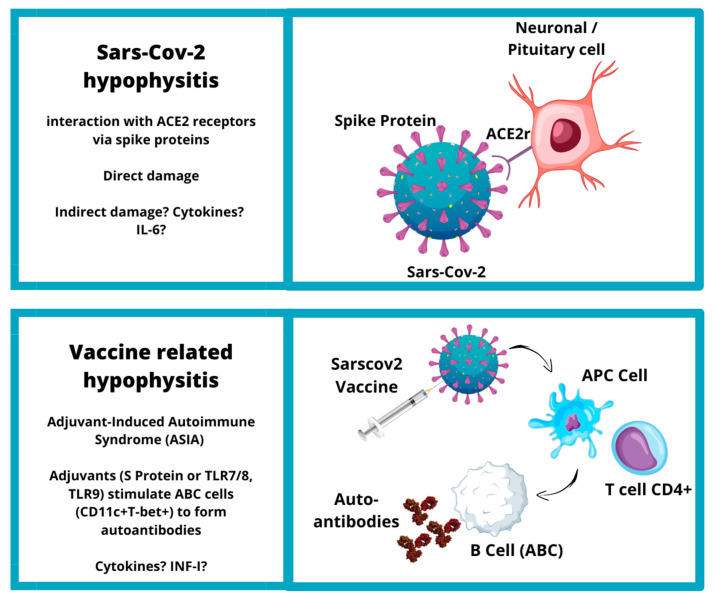
Etiopathogenesis of SARS-CoV-2 and vaccine-related hypophysitis (ACE2: angiotensin-converting enzyme 2; IL-6: interleukin 6; TLR: tall cell receptor; INF-I: interferon I).

The primary etiopathogenetic hypothesis suggests that SARS-CoV-2 infiltrates through systemic vascular dissemination and across the cribriform plate of the ethmoid bone. The virus enters the central nervous system because of its distinct interaction with angiotensin-converting enzyme 2 (ACE2) receptors via spike proteins binding the capillary endothelium. Once within the neuronal tissues, its interaction with ACE2 receptors (that are expressed also in the hypothalamus and pituitary glands) can trigger a cycle of viral budding, which is accompanied by pituitary damage and inflammation [80]. The anterior pituitary, located outside the brain–blood barrier, may also be directly affected by SARS-CoV-2. The transport of the COVID-19 virus to the brain and pituitary via the cribriform plate near the olfactory bulb may provide an additional route that allows the virus to reach and impact the brain [76,78,80] (Figure 3).

Thus, the proinflammatory properties leading to hypophysitis could be accounted for via the direct targeting of hypothalamic and pituitary tissues due to high expression of ACE2 receptors [12,76,80]. However, theoretically, the effect of SARS-CoV-2 on the function of the hypothalamic–pituitary axis could occur indirectly through inflammation and the release of cytokines [77,81]. The exact mechanism remains ambiguous; more crucially, there is no evidence to date of a direct mechanism. Reports have indicated a lack of immunoreactivity for viral protein in pituitary cells [82,83]. Histological examinations found no evidence of COVID-19-specific changes in the pituitary glands of 50 fatal COVID-19 cases [82]. 

It is worth mentioning that the onset of arginine vasopressin (AVP) deficiency was also reported during or shortly after COVID-19 infection in several cases, which suggests a potential causal relationship [9,76,84]. Hypophysitis or an immune-mediated reaction are possible mechanisms to explain AVP deficiency in this context, although the reported cases so far are too few to draw any substantial conclusions. An inverse correlation between serum sodium and serum interleukin 6 (IL-6) levels (with IL-6 being a crucial cytokine involved in the fatal outcomes of COVID-19 due to a cytokine storm) provides a possible pathogenic link. Additionally, IL-6 can induce vasopressin secretion, and the correction of hyponatremia after treatment with IL-6 receptor antibody (tocilizumab) has been reported [85,86].

At present, there are no predictive markers that could differentiate COVID-19 cases most likely to impact either the hypothalamus or the pituitary [77]. Lastly, since the resolution of hypophysitis is usually achieved with the administration of glucocorticoids, widely used for treating patients with COVID-19, it could be hypothesized that such therapy may result in a reduced or stable incidence of hypophysitis in patients with COVID-19 [76].

### 5.2. SARS-CoV-2 Vaccine and Hypophysitis

Autoimmune syndromes triggered by vaccinations are uncommon, and they generally fall under the scope of adjuvant-induced autoimmune syndrome (ASIA) [9,10,11,87,88]. Understanding these autoimmune syndromes offers new insights into the intricate interplay among the host, the immune system, and new vaccines. The available data on endocrine complications associated with the SARS-CoV-2 vaccine are scant. Nonetheless, isolated cases of hypophysitis have been documented [12,88].

Adjuvants, substances employed to amplify and prolong the immune response, are invaluable in refining vaccines. However, in genetically predisposed individuals, exposure to adjuvants may occasionally trigger polygenic autoimmune diseases. The immune disruption in these instances is primarily due to molecular mimicry, initiating polyclonal activation of B lymphocytes [81,88]. A review of the adjuvants used in COVID-19 vaccines revealed that elements such as aluminum salts, emulsions, oils, S protein, Toll-like receptors, four lipids of the mRNA vaccine, and polyethylene glycol might incite an immune response in susceptible individuals [81].

The distinctive characteristics of the S protein, a vaccine-encoded antigen, could be responsible for vaccination-related adverse effects. This is due either to molecular mimicry with human proteins or its role as an ACE2 ligand. When introduced into circulation through vaccination, the S protein may heighten autoimmune disease risk in predisposed individuals [10,76,81,88] (Figure 3). 

With regard to the immune system’s role, the involvement of age-associated B cells (ABC) in the immune response triggered by the SARS-CoV-2 vaccine has been suggested. These ABC cells (CD11c^+^T-bet^+^) increase with age in healthy individuals and are often present early in autoimmune diseases and infections. They are characterized by their production of immunoglobulin G, enhancement of antigen presentation to T cells, and germinal center formation. Another key trait of these ABC cells is their propensity to trigger a hyper-response upon stimulation with Toll-like receptor 7 (TLR7) signaling, thereby generating autoreactive antibody-secreting plasmablasts. mRNA/DNA SARS-CoV-2 vaccines use TLR7/8 and TLR9 agonists as “adjuvants”, which may stimulate this ABC subgroup to form autoantibodies and post-vaccine autoimmune syndromes. The activation of TLR7 and TLR9 can lead to the production of interferon I, a crucial cytokine for the development of autoimmune reactions [10,87,88].

Despite these proposed etiopathogenic causes, the precise mechanisms underpinning post-COVID-19 vaccine autoimmune syndromes remain to be clarified. Pituitary diseases induced by the SARS-CoV-2 vaccine can present with symptoms similar to classical post-vaccination syndrome (headache, asthenia, etc.). This overlap may result in some cases of pituitary-induced diseases going unnoticed, especially if clinicians do not anticipate and probe for their presence [77,81]. Due to the limited number of documented vaccine related hypophysitis cases, we are currently lacking reliable predictive factors for its onset. Consequently, we recommend implementing screening procedures for hypophysitis in patients presenting with symptoms including headaches, sudden fatigue, hypotension, polyuria, polydipsia, electrolyte imbalances, and abnormalities in menstrual cycles and fertility.

## 6. Personalized Medicine in the Treatment of Hypophysitis: Is There a Place?

Glucocorticoids (GCs) are the cornerstone in managing primary hypophysitis, given their targeted action on inflammation. However, the occurrence of spontaneous resolution of pituitary infiltration, with or without enduring pituitary dysfunction, is a frequently observed phenomenon [4,15]. The absence of randomized control trials makes it uncertain whether GC therapy truly leads to a superior recovery of pituitary function compared to mere observation. Owing to the vast array of GC side-effects, careful evaluation of the risks and benefits is crucial before initiating treatment, especially in mild hypophysitis cases [4,22].

The natural trajectory of hypophysitis, characterized by acute and chronic phases, informs the management, treatment, and follow-up of both PAH and secondary hypophysitis. In the acute phase of PAH, a multidisciplinary assessment of the risk/benefit ratio should be conducted to determine whether to propose glucocorticoid immunosuppressive therapy, which could potentially recover pituitary function and prevent the progression to persistent hypopituitarism and empty sella syndrome. In the chronic phase, where irreversible alterations have set in, anti-inflammatory therapy may not influence radiologic or hormonal outcomes [4,22].

The response rate to GCs in primary hypophysitis varies widely, ranging from 20% to greater than 95%, with either partial or complete hormonal and radiographic response. Noticeably, the improvement in endocrine function is less frequent than the reduction in pituitary mass, which could achieve nearly a 75% response rate. Early initiation of GC therapy has been associated with improved hormonal recovery [89,90,91].

For IIHs, coordinated decision making between the oncologist and endocrinologist is vital to ensure the best therapeutic approach, thereby averting life-threatening situations and enhancing patient outcomes [1,14]. Physiological hormone replacement with hydrocortisone and/or thyroxine is advised. It may be appropriate to delay immunotherapy in the acute phase until the patient’s condition stabilizes. Nevertheless, discontinuation does not appear to enhance pituitary outcomes and may result in cancer progression [14,92]. High-dose GCs should be reserved for patients with severe symptoms of mass effect, visual loss, or adrenal crisis [93]. Interestingly, the occurrence of an immune-related event seems to predict better overall survival compared to patients without such an event [94].

Studied prednisone doses for treating primary hypophysitis are wide-ranging, from an initial dose of 20 mg/day to 1 g of pulse therapy with methylprednisolone. Given the rarity of this condition and the lack of extensive randomized prospective clinical trials, the precise dose, duration, and even the indication for GC therapy remain debatable [4,42]. Some authors argue for the use of pulse regimens of high-dose methylprednisolone (120 mg/g) administered intravenously [90]. Lower GC doses may also prove effective. A prospective study contrasted 12 patients treated with 50 mg prednisone for 3 months with a slow taper against eight patients managed by observation. At the end of a 2 year follow-up, a higher percentage of prednisone-treated patients (58.3%) improved their pituitary function compared to untreated patients (25%), and 66% of treated versus 25% of untreated patients showed radiographic improvement [42].

In cases refractory to GCs, immunosuppression with rituximab, azathioprine, or methotrexate could be considered as GC-sparing options. Among these, azathioprine has been most extensively studied and appears to be more beneficial for mass reduction than for hormonal improvement [22]. Similar outcomes have been noted with methotrexate and mycophenolate mofetil [4,22].

Surgery may be utilized not only for decompressing the optic chiasm and GC-resistant cases but also to affirm the diagnosis in ambiguous cases. Both fractionated radiotherapy and stereotactic radiosurgery have demonstrated success in a handful of patients requiring multimodal therapy [4,22].

In the context of COVID-19, glucocorticoids, the standard treatment for hypophysitis, are widely used in managing severe COVID-19 cases. Thus, one might hypothesize that this therapy could result in a reduced or stable incidence of hypophysitis in patients with COVID-19. Importantly, recent research has suggested potential beneficial effects in severe COVID-19 for both dexamethasone and methylprednisolone, likely by inhibiting SARS-CoV-2 entry into cells via ACE2 binding. The use of high-dose potent exogenous glucocorticoids could lead to the suppression of the hypothalamic–pituitary–adrenal axis and subsequent adrenal insufficiency, depending on the dose, treatment duration, and individual drug sensitivity [76,77,95]. Patients with known hypopituitarism, specifically those with adrenal insufficiency, face an increased mortality risk compared to the general population, with infections being one of the primary causes of death. However, optimal patient education and heightened awareness among medical staff about the potential for adrenal crisis during an infection can significantly mitigate this risk [77].

The efficacy of the SARS-CoV-2 vaccine can be diminished in patients undergoing various immunosuppressive therapies, particularly glucocorticoids. However, there are no such data available for patients on glucocorticoid replacement. A two- to threefold increase in the usual dose is recommended if the patient exhibits any symptoms following COVID-19 vaccination, as acute adrenal insufficiency can sometimes occur. In a prospective study, up to 8% of patients required a doubling of the oral glucocorticoid dose, especially after the second vaccine dose, but no parenteral administration was required [77,81,88,96,97].

Better understanding of the etiopathogenesis and molecular mechanism of hypophysitis can lead to more effective and personalized treatment strategies [15]. Unfortunately, there is a paucity of data on personalized therapeutic strategies in the context of hypophysitis. For patients diagnosed with PAH, the endorsement of immunosuppressive treatment, involving glucocorticoids, azathioprine, or mycophenolate mofetil, is advisable, particularly in younger individuals and those suffering from partial or complete hypopituitarism, diabetes insipidus, or neurological or ophthalmic manifestations. It is essential, however, to exclude major contraindications to these treatments prior to initiation. Interestingly, a significant subset of patients might witness the restoration of pituitary functionality following the administration of immunosuppressive therapies, particularly in instances of central hypogonadism and growth hormone deficiency [42]. Rituximab, an anti-CD20^+^ agent, has demonstrated utility in diseases predominantly involving B lymphocytes and in recurrent IgG4-related diseases [98].

Looking toward the future, there is potential for comprehensive investigation into the discovery and identification of influential factors and biomarkers. These could play a critical role in guiding the selection of the most suitable therapeutic interventions for patients suffering from hypophysitis. The identification of new etiopathogenic causes and autoantigens could significantly facilitate the development of targeted therapies. For instance, the discovery of specific autoantigens could lead to the formulation of monoclonal antibody therapies, providing a more personalized and potentially effective treatment for this condition.

## 7. Conclusions

Hypophysitis is an intricate pathological entity. While it was once considered an extremely rare occurrence, a more precise definition and a deeper comprehension of its pathogenesis have illuminated the likelihood of its more frequent occurrence than previously estimated. Furthermore, the escalating utilization of ICIs for cancer treatment, coupled with hypophysitis related to post-viral conditions and vaccines, specifically in relation to COVID-19, might be contributing to an increased incidence.

Looking ahead, novel insights into the etiopathogenesis, as well as the genetic and molecular mechanisms, of hypophysitis could be beneficial for diagnosis and therapeutic interventions. A sustained effort to identify reliable biomarkers and pituitary autoantibodies is crucial to formulate accurate, noninvasive diagnostic tests and new targeted therapies.

## Figures and Tables

**Table 1:** Summary of hypophysitis types, etiology, and histology features.

	Etiology	Sex	Histology	References
Autoimmune	APA and AHA	>F	Lymphocitic infiltration	[4]
Lymphocytic	Autoimmune, pregnancy, and peripartum T-cell-mediated	F (30 years) pregnancy and peripartum M (40 years) autoimmunity	Lymphocitic infiltration, plasmacells, histocites, and fibrosis	[4,5]
Granulomatous	Primary: idiopathic (APA? cronic LH?) Secondary: sarcoidosis, granulomatosis with polyangitiis, langherans cells histocystosis, tubercolosis, Wegener’s granulomatosis, Erdheim–Chester disease, Crohn’s disease, Takayasu arteritis, Cogan’s syndrome, and other vasculites	>F	Multinucleated giant cells, histiocytes, lymphocytes, granulomas	[4,5]
Xanthomatous	Local: hemorrage, cyst ropture, craniopharingioma Xanthogranulomatous: chronic?	>F (40 years)	CD68-positive foamy, macrophages, colesterol clefts, hemosiderin	[4,5]
Necrotizing	Unknown (autoimmune?)	>F very rare	Extensive necrosis, lymphocytes, plasmacytes, few eosinophlis	[4,5]
IgG4-related	IgG4 disease, POMC, and GH autoantigen?	>M (50–70 years)	>10 high-power field, IgG4 plasma cells, fibrosis	[4,5]
ICI induced	Anti-CTLA4 and anti-PD1/PDL1	>M	Diffuse infiltration with lymphocytes and macrophages	[2]
Paraneoplastic	Anti-Pit1, anti-POMC, or anti-ACTHEctopic expression of ACTH, Pit1, or other	?	Lymphocitic infiltration	[7]

F: female; M: male; APA: anti-pituitary antibodies; AHA: anti-hypothalamus antibodies; LH: lymphocytic hypophysitis; GC: glucocorticoid; ICI: immune checkpoint inhibitor; IgG4: immunoglobulin G4; POMC: pro-opiomelanocortin; GH: growth hormone; CTLA4: cytotoxic T-lymphocyte-associated protein 4; PD1: programmed cell death protein-1; PDL1: programmed death ligand-1; ACTH: adrenocorticotropic hormone; Pit1: pituitary transcription factor 1; CD68: cluster of differentiation 68.

## Data Availability

No new data were created or analyzed in this study. Data sharing is not applicable to this article.
